# Optimized reconstruction of the absorption spectra of kidney tissues from the spectra of tissue components using the least squares method

**DOI:** 10.1002/jbio.202300466

**Published:** 2024-02-06

**Authors:** Maria R. Pinheiro, Luís E. Fernandes, Isa C. Carneiro, Sónia D. Carvalho, Rui M. Henrique, Valery V. Tuchin, Hélder P. Oliveira, Luís M. Oliveira

**Affiliations:** ^1^ Telecommunications and Multimedia Center (CTM) Institute for Systems and Computer Engineering, Technology and Science (INESC TEC) Porto Portugal; ^2^ Department of Electrical and Computer Engineering Faculty of Engineering, Porto University Porto Portugal; ^3^ Department of Pathology and Cancer Biology and Epigenetics Group Portuguese Oncology Institute of Porto Porto Portugal; ^4^ Department of Pathological, Cytological and Thanatological Anatomy Polytechnic of Porto – School of Health (ESS) Porto Portugal; ^5^ Department of Pathology Santa Luzia Hospital (ULSAM) Viana do Castelo Portugal; ^6^ Department of Pathology and Molecular Immunology Institute of Biomedical Sciences Abel Salazar, Porto University Porto Portugal; ^7^ Science Medical Center Saratov State University Saratov Russia; ^8^ A. N. Bach Institute of Biochemistry RC “Biotechnology of the Russian Academy of Sciences” Moscow Russia; ^9^ Laboratory of Laser Molecular Imaging and Machine Learning Tomsk State University Tomsk Russia; ^10^ Department of Computer Science Faculty of Science, Porto University Porto Portugal; ^11^ Physics Department Polytechnic of Porto – School of Engineering (ISEP) Porto Portugal

**Keywords:** absorption spectrum reconstruction, kidney absorption spectra, quantification of components contents, spectral diagnostic information, tissue component identification

## Abstract

With the objective of developing new methods to acquire diagnostic information, the reconstruction of the broadband absorption coefficient spectra (*μ*_a_[*λ*]) of healthy and chromophobe renal cell carcinoma kidney tissues was performed. By performing a weighted sum of the absorption spectra of proteins, DNA, oxygenated, and deoxygenated hemoglobin, lipids, water, melanin, and lipofuscin, it was possible to obtain a good match of the experimental *μ*_a_(*λ*) of both kidney conditions. The weights used in those reconstructions were estimated using the least squares method, and assuming a total water content of 77% in both kidney tissues, it was possible to calculate the concentrations of the other tissue components. It has been shown that with the development of cancer, the concentrations of proteins, DNA, oxygenated hemoglobin, lipids, and lipofuscin increase, and the concentration of melanin decreases. Future studies based on minimally invasive spectral measurements will allow cancer diagnosis using the proposed approach.
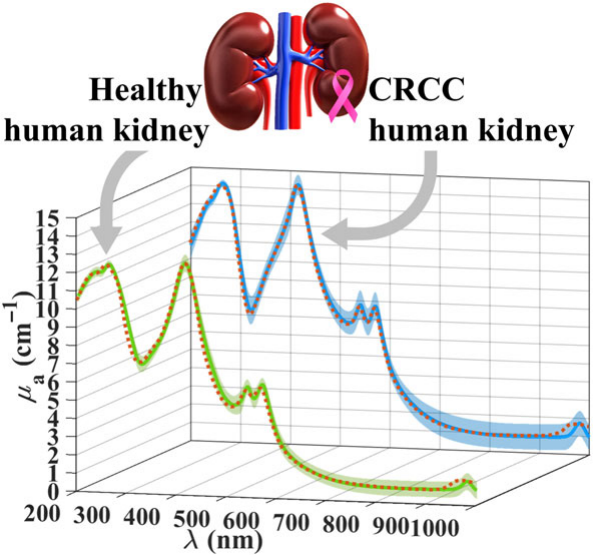

## Graphical abstract

The absorption coefficient spectra of healthy and cancerous kidney tissues were reconstructed from the absorption spectra of tissue components using constant weights. Both reconstructions allowed to calculate the concentrations of the tissue components and a comparison between the healthy and chromophobe renal cell carcinoma kidney showed an increase in lipids, blood, and DNA. The variations observed for melanin and lipofuscin led to the idea of melanolipofuscin granule formation when cancer develops.

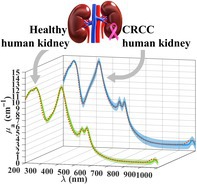

## 1 INTRODUCTION

The urinary system has the crucial functions of regulating, filtering, and excreting toxins, metabolic waste products, and even excess of ions that circulate in the bloodstream [1]. The kidney is one of the most important organs of the urinary system and several pathologies are associated with this organ [2, 3]. Renal cell carcinoma (RCC) is thought to arise from the tubules in the kidney [4, 5] and comprises several subtypes that differ in tumor aggressiveness and the risk of metastasis, with chromophobe renal cell carcinoma (CRCC) being the third most common subtype [6, 7]. Due to the importance of early cancer diagnosis, several ways of diagnosing this pathology have been studied and developed [8]. The majority of renal tumors are detected incidentally following investigations for nonspecific symptoms or other abdominal diseases. Once detected, the evaluation of renal tumors usually relies on cross‐sectional imaging modalities, such as ultrasonography, computed tomography, and/or magnetic resonance imaging. Additionally, percutaneous image‐guided biopsy is performed in selected cases to provide further information to choose the most suitable medical and/or surgical treatment strategy. Renal tumor biopsy is a procedure that removes a sample of tumoral tissue to be analyzed “ex vivo” in a laboratory, being the only method to provide accurate details on histological subtyping and grade [9]. With increasing incidental findings of small renal masses, a need for a more conservative approach is pressing. Thus, in selected cases, nonsurgical ablation of these tumors is deemed to be performed but this requires robust information concerning diagnosis, as some renal cell tumors are aggressive despite their size and require a more radical treatment. Because biopsy has also limitations, owing to the well‐known tumor heterogeneity, the ability to characterize the whole tumor using a noninvasive or minimally invasive procedure would provide an assertive characterization of the biological potential of the tumor. This would require an in vivo approach.

Optical technologies play an important role in the diagnosis of cancer and can be used in vivo to provide an early‐stage disease diagnostic [10]. Research studies with biological tissues, blood, and their components use a variety of optical techniques, including fluorescence spectroscopy, visible and near‐infrared (NIR) spectroscopy, or even optical coherence tomography [11]. The estimation of the spectral optical properties of biological tissues is of great interest for the application of such techniques since they quantify the light scattering, the light absorption, and the light penetration depth within a spectral range of interest for those optical technologies. An additional advantage of estimating the spectral optical properties of tissues is that they are unique for each tissue, allowing the discrimination between healthy and pathological conditions [12]. The spectral absorption coefficient (*μ*_a_[*λ*]), in particular, provides direct information for the identification of the tissue components and their contents in a tissue [12, 13]. For any tissue, once its *μ*_a_(*λ*) is obtained, the identification of the absorption bands in that spectrum allows to recognize the components that the tissue contains and to acquire information about their contents from the absorption ratios detected at those bands [12]. It has been reported that when cancer develops, the contents of DNA, proteins, and blood increase with respect to the healthy tissue [12, 14]. Consequently, the quantification of the concentrations of these tissue chromophores in healthy tissues and tissues with different stages of cancer development from the analysis of their *μ*_a_(*λ*) has a great interest for the development of new noninvasive or minimally invasive diagnostic protocols. Once such data is obtained from tissue biopsies, the combination of diffuse reflectance (*R*_d_) spectra with machine learning (ML) algorithms to estimate the *μ*_a_(*λ*) can be used for in vivo early‐stage diagnosis [15]. Considering the large number of known cancers, such is a multitasking research, which involves the determination of the broadband *μ*_a_(*λ*) of various tissues, both in healthy and with different stages of cancer development from tissue biopsies and the subsequent development of various ML algorithms to estimate those spectra from noninvasive or minimally invasive *R*_d_ spectra. This study is focused on the first stage of this multi‐tasking research for the case of the CRCC.

The *μ*_a_(*λ*), or the other spectral optical properties of a tissue, cannot be measured directly. Traditionally, they are estimated through inverse computer simulations, where experimental spectral data collected from the tissue under study are used as input [16]. Such simulations are based on solving the radiation transport equation for nonhomogeneous media and, since they were developed with various methods, they differ in their designations and available algorithms, such as the Kubelka–Munk, the Monte Carlo, and the Adding‐Doubling simulation codes [16]. The main disadvantage of these inverse simulations is that they only estimate a set of optical properties for a single wavelength, *λ*, at a time, which makes the estimation of the spectral optical properties time‐consuming and computationally dispendious [12].

Another way to estimate the spectral optical properties of tissues is through direct calculations, which are based on the photon diffusion approximation [12, 17]. As previously mentioned, the broadband *μ*_a_(*λ*) is one of the most important optical properties since it allows to identify the chromophores that the tissues contain, and even their concentrations. When the *λ* of the exciting light corresponds to an absorption band of any of the biological components in a tissue, light absorption occurs [18]. When experimental spectra are acquired from an ex vivo tissue sample, and considering that light that irradiates the sample can only be transmitted, reflected, or absorbed, the *μ*_a_(*λ*) of the tissue can be directly calculated at once from the total transmittance (*T*_t_) and total reflectance (*R*_t_) spectra measured from the sample [19]:

(1) $$\mu_{a}\left(\lambda \right)=\frac{\left[1-\left(T_{t}\left(\lambda \right)+R_{t}\left(\lambda \right)\right)\right]}{d},$$

where *d* represents the thickness of the sample used in the measurements of the experimental spectra. The use of *T*_t_ (*λ*) in Equation (1) implies that this calculation to obtain *μ*_a_(*λ*) needs invasive measurements, which are impracticable for the in vivo situation, but can be used for rapid ex vivo examination of tissue biopsies. After the *μ*_a_(*λ*) is calculated with Equation (1), the combination of ML algorithms with noninvasive or minimally invasive spectral measurements, such as the *R*_d_ spectra can be used to predict the *μ*_a_(*λ*) of healthy and pathological tissues, as reported in previous studies [15, 20].

As a result of these studies, the application of optical methods to obtain cancer discriminating information has great potential and interest. Biological tissues contain different components, such as DNA, hemoglobin, water, lipids, and pigments, meaning that the global broadband *μ*_a_(*λ*) of a tissue can be expressed as a weighted sum of the *μ*_a_ spectra of the various components [13, 21]. Therefore, by digitally reconstructing the *μ*_a_(*λ*) of a tissue, the contribution of each of the biological components in the tissue can be quantified, and differentiated chromophore quantities that are obtained between normal and cancerous tissues can be useful for new diagnostic protocols. With the purpose of evaluating potential discriminating information between the normal and cancerous (CRCC) conditions of kidney, this study consisted of reconstructing the experimental *μ*_a_ spectra of those tissues as a weighted sum of the absorption spectra of some tissue components (*μ*_a*i*_ [*λ*]) [13, 21]:

(2) $$\mu_{a}\left(\lambda \right)=\sum_{i}w_{i}\times \mu_{\mathrm{ai}}\left(\lambda \right),$$

where *w*_*i*_ are the weights or contributions of each component to the absorption spectrum of the kidney tissues. Before performing the reconstruction of the *μ*_a_(*λ*) of the kidney tissues, the appropriate *w*_*i*_ values need to be obtained. The most simple way to obtain such values is by trial‐and‐error, where the various *w*_*i*_ values are fine‐tuned to lead to an optimized reconstruction of the experimental *μ*_a_(*λ*) of the kidney tissues. Such an approach to obtain the optimal *w*_*i*_ values is somehow time‐consuming and it may not produce the optimized reconstruction. As an alternative to optimize the reconstruction of the *μ*_a_(*λ*) there are some methods, such as the least squares method [22, 23], the non‐negative matrix factorization [24], or the multivariate curve resolution alternating least squares method [25]. In this work, we selected the least squares method due to its simplicity which makes the retrieval and the subsequent study of the weights an easier task. Furthermore, when compared with other ML algorithms, the least squares method requires less data to converge into a solution. The implementation of such a method is described in Section 2.

## 2 MATERIALS AND METHODS

Since this study concerns the reconstruction of the experimental *μ*_a_(*λ*) of healthy and cancerous kidney tissues, several tasks were performed, initiating with the tissue collection from patients and ending with the final reconstructed spectra. The following subsections describe such tasks, starting with Section 2.1, where a description is made for the methods used to collect the tissues from patients and to prepare the samples for the spectral measurements. Section 2.2 describes the setups and methodology used to acquire the experimental spectra from the tissue samples and Section 2.3 describes the direct calculation of the *μ*_a_ spectra from the experimental measurements. A description of the methodology adopted for the reconstruction of the *μ*_a_ spectra is made in Section 2.4.

### 2.1 Tissue collection and sample preparation

**FIGURE 1:**
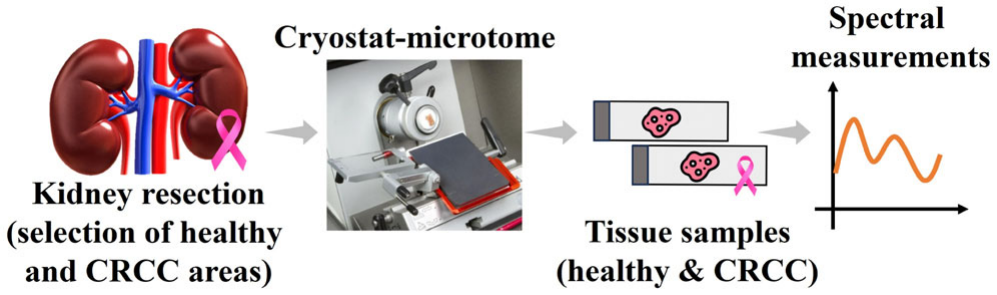
Collection and preparation of tissue samples of healthy and chromophobe renal cell carcinoma (CRCC) kidney tissues to be used in the spectral measurements.

The tissue samples used in this study were collected from kidney resections, which were obtained from 10 patients under surgical treatment at the Portuguese Oncology Institute of Porto (IPO‐Porto), Portugal. Previous imaging exams of these patients have identified the presence of renal masses, which were surgically removed. The collected samples contained both healthy and tumor areas, which were separated into two groups that contained fragments from both conditions. The first group was used for further analysis to identify the cancer subtype and grade, while the second group was used to prepare the samples for this study. Histopathological analysis of the tumor confirmed that it was CRCC. The patients from whom the tissue resections were collected have previously signed a written consent allowing for the use of the collected tissues in research and diagnostic procedures and this study was approved by the Ethics Committee of IPO‐Porto. A cryostat‐microtome (model CM1850 UV from Leica™, Wetzlar, Germany) was used to prepare 10 samples from the healthy areas and another 10 from the tumor (CRCC) areas of the collected resections. All these samples were prepared with an approximated circular form (*ϕ* = 1 cm) and a uniform thickness of 0.5 mm. In total, 20 kidney samples were used in the spectral measurements, consisting of 10 pairs of healthy/CRCC samples collected from each of the 10 patients. After preparing the samples, they were immersed in saline for 10 min to simulate their natural hydration, before being used in the spectral measurements. The steps for the sample collection and preparation are represented in Figure 1.

### 2.2 Spectral measurements

**FIGURE 2:**
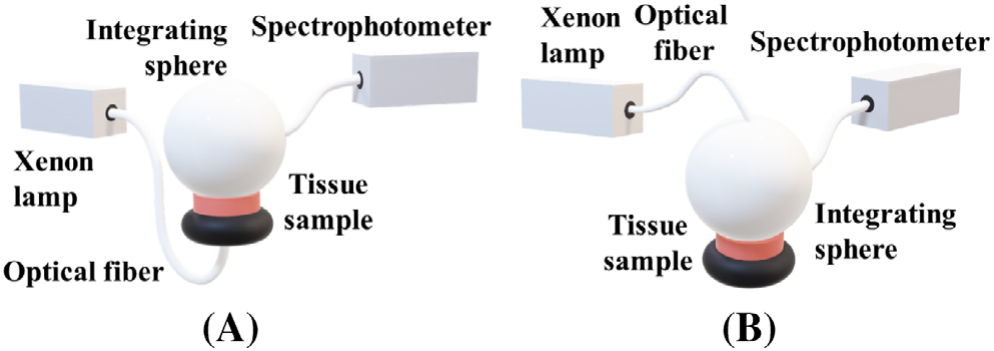
Experimental setups to measure the *T*_t_ (A) and *R*_t_ (B) spectra.

To acquire the necessary experimental spectra to calculate the *μ*_a_(*λ*) of the kidney tissues, two experimental setups were used. These setups, which are represented in Figure 2, allow the measurement of the *T*_t_ and *R*_t_ spectra of tissue samples, as described by Martins et al. [26]. Each of the samples used in this study, 10 from the healthy and 10 from the CRCC areas of the kidney resections were submitted to measurements with both setups, considering a spectral range between 200 and 1000 nm.

Considering both setups presented in Figure 2, each of the kidney samples was placed, one at a time, at the sample port of the integrating sphere (AvaSphere‐50 from Avantes™). A broadband, high‐power, and pulsed xenon lamp (Avalight‐XE‐HP from Avantes™) irradiated the tissue sample through an optical fiber cable. Such irradiation was made from below the sample in the *T*_t_ setup, and from the top of the sphere at 8° with the vertical axis of the sphere in the *R*_t_ setup. The beam that was transmitted or reflected by the sample was submitted to the integration process, by being reflected several times at the internal surface of the sphere. Another optical fiber cable collects the integrated beam that exits the integrating sphere, to deliver it to the spectrophotometer (AvaSpec‐2048‐USB2 from Avantes™) to register the *T*_t_ or *R*_t_ spectrum. After acquiring all the experimental spectra, calculations, as described in Section 2.3, were performed to obtain the *μ*_a_(*λ*) for all the samples under study.

### 2.3 Direct calculation of the *μ*_a_ spectra of both kidney conditions

To calculate the *μ*_a_ spectra for all samples from the measured T_t_ and R_t_ spectra, Equation (1) was used. Such calculation was made for all samples under study: 10 from healthy kidney and 10 from CRCC kidney. The individual *μ*_a_(*λ*) were later used to obtain the mean and standard deviation (SD) for the absorption spectrum of the healthy and CRCC kidney tissues. Such calculations were performed to allow the representation of statistical data for the two conditions of the kidney, and to serve as reference for the reconstruction procedure that was later performed as described in Section 2.4.

### 2.4 Reconstruction of the *μ*_a_ spectra of both kidney conditions

Due to the fact that the broadband *μ*_a_(*λ*) of any tissue can be described as a weighted sum of the *μ*_a*i*_(*λ*) of tissue components [13], the main objective of this study was to perform the reconstruction of the mean absorption spectra of the healthy and CRCC kidney tissues to access additional information that can be used for diagnostic purposes. A first analysis of the calculated *μ*_a_ spectra of both kidney conditions (Figure 6, in Section 3) revealed the presence of proteins, DNA, hemoglobin, and water. It also revealed the presence of a *λ*‐decreasing baseline, which indicates that such broadband decreasing absorption results from the accumulation of melanin and lipofuscin [27, 28]. Such pigment accumulation has been already observed for other tissues and in some cases associated with the tissue aging process [26], or the development of diseases [17, 29]. In the case of the kidney, melanic pigments can occur at a small scale as a product of melanocytes. The precursors of melanocytes are formed in the neural crest during embryogenesis, from where they migrate into different organs, including ectopic places such as the developing genitourinary tract [30, 31]. These pigments have also been reported in some rare kidney tumors, namely microphthalmia‐associated transcription family translocation RCC, eosinophilic solid, and CRCC, and perivascular epithelioid cell tumor [32, 33, 34]. Lipofuscin pigment can occur virtually in every organ since it is mainly the product of the oxidation of fatty acids that occurs as a result of the degradation and turnover of cellular components [35]. Its deposition in cells can be associated with a decline in the lysosomal degrative capacity or abnormalities in lipid peroxidation. In the kidney, lipofuscin deposits have been related to different physiological and medical conditions [36].

**FIGURE 3:**
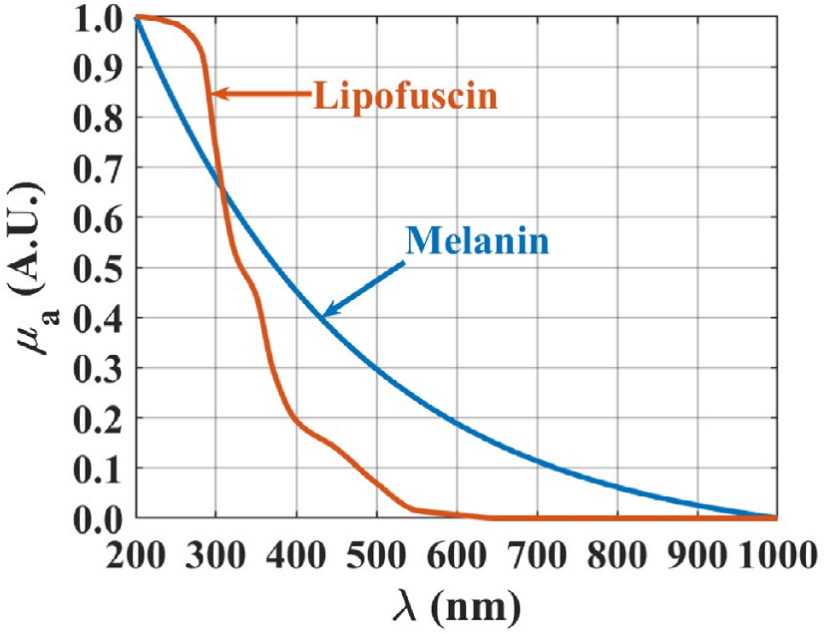
*μ*_a_(*λ*) of melanin [27] and lipofuscin [28], between 200 and 1000 nm.

**FIGURE 4:**
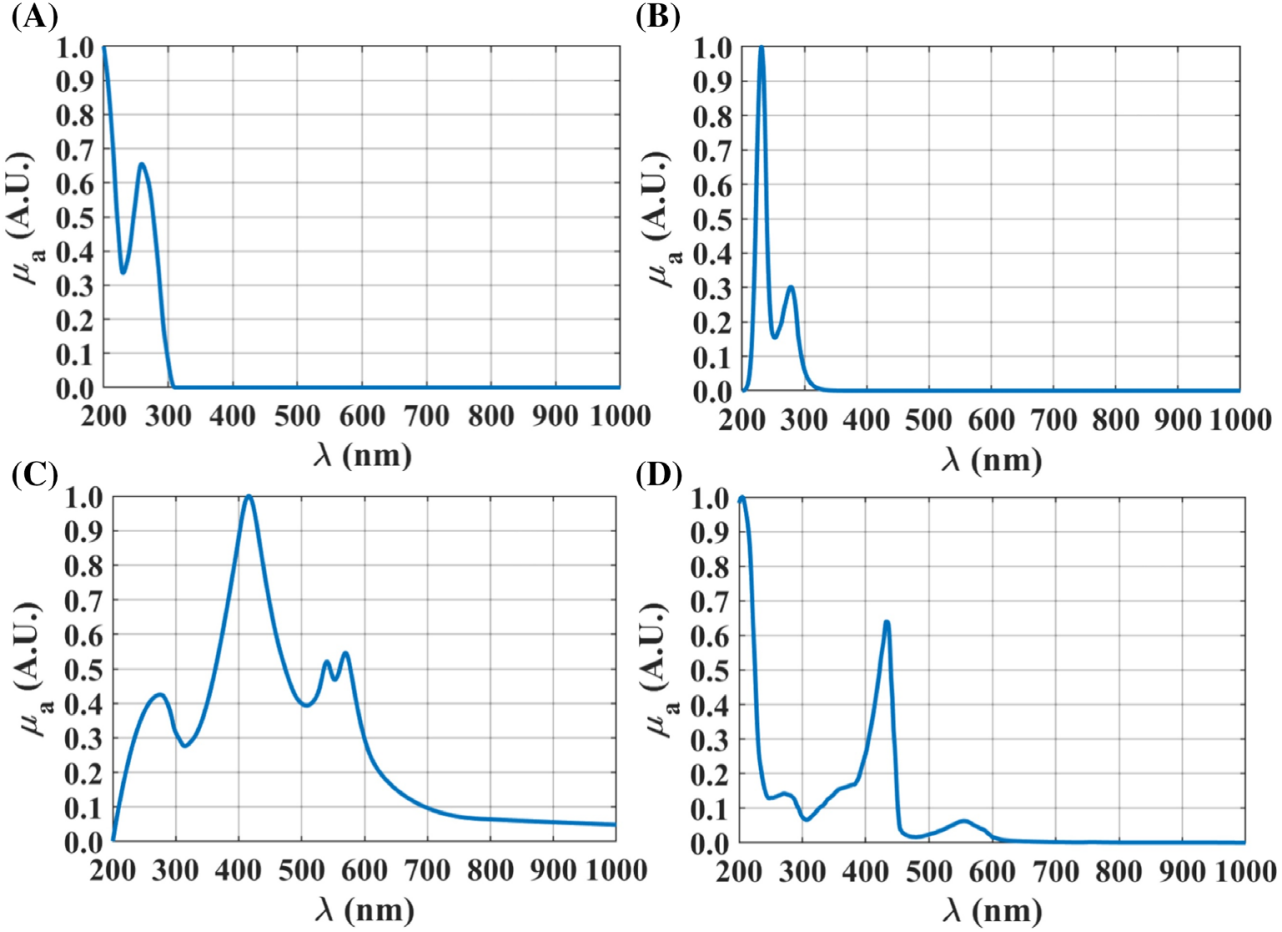
*μ*_a_ spectra of DNA (A) [38], proteins (B) [38], oxygenated hemoglobin (C), and hemoglobin (D) [39], between 200 and 1000 nm.

**FIGURE 5:**
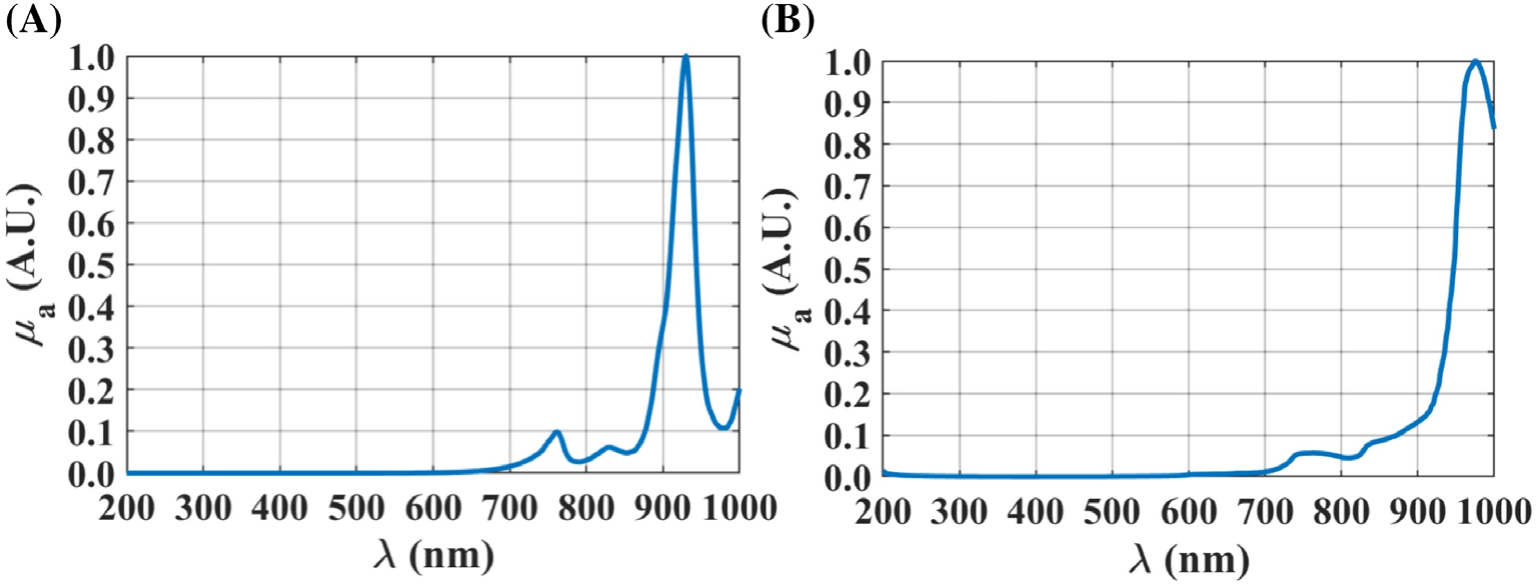
*μ*_a_ spectra of lipids (A) [41] and water (B) [42, 43, 44], between 200 and 1000 nm.

A previous study with human colorectal mucosa tissues [37] also showed that tissues with colorectal cancer tumors present higher contents of lipids than healthy tissues. Considering this identification of possible components in the healthy/CRCC kidney tissues, we selected the absorption spectra of melanin, lipofuscin, proteins, DNA, oxygenated hemoglobin (HbO_2_), and deoxygenated hemoglobin (Hb), lipids, and water to be used in the reconstruction of the *μ*_a_(*λ*) of both kidney tissues with Equation (2). Those spectra are presented in Figures 3, 4, 5 between 200 and 1000 nm, after being normalized between 0 and 1 for a better comparison.

According to Figure 3, both pigments present higher absorption values in the deep ultraviolet. The curves presented in Figure 3 were reconstructed from data presented in Ref. [27] in the case of melanin, and from data presented in Ref. [28] in the case of lipofuscin.

Considering the other chromophores that we have selected to use in the reconstruction of the *μ*_a_(*λ*) of both kidney conditions, DNA, proteins, and both forms of hemoglobin also present absorption bands in the ultraviolet (UV), as represented in Figure 4.

The graphics presented in Figure 4 were reconstructed from graphical or numerical data presented in some publications. The ones for DNA and proteins were reconstructed from graphical data in Ref. [38], while the one for Hb was reconstructed from numerical data in Ref. [39]. For the case of HbO_2_, although data to construct its absorption spectrum is available [40], it corresponds to pure hemoglobin diluted in water, and not to the case of HbO_2_ in the blood inside the microveins of the kidney tissues used in this study. For this reason, the absorption spectrum for HbO_2_ that is presented in Figure 4C was measured in our lab from an aqueous solution of fresh human blood that was collected from a human volunteer. That spectrum was the one that provided the best match in the reconstruction of both *μ*_a_ spectra of the kidney tissues.

Considering now the case of lipids and water, they present absorption bands located in the near‐infrared, as represented in Figure 5.

The absorption spectrum of lipids that is presented in Figure 5A was created with numerical data from Ref. [41] and the one for water that is presented in Figure 5B was constructed from graphical data from Refs. [42, 43, 44], which correspond to different spectral ranges. In the case of water, and considering the data collected from Refs. [42, 43], although its absorption spectrum presents a decreasing behavior from 200 to 600 nm, the absorption values in that spectral range are much lower than the ones seen in the near‐infrared. For this reason, in Figure 5B, water seems to have zero absorption between 200 and 600 nm. In the case of lipids, no data for the absorption coefficient is available for *λ* < 429 nm. Considering the numerical data from Ref. [41], lipids have a decreasing absorption from 429 to 620 nm, with values much lower than the ones observed in the near‐infrared. For this reason, when constructing the *μ*_a_(*λ*) of lipids that is presented in Figure 5A, we considered that lipids have zero absorption between 200 and 620 nm. This approximation will not influence the reconstruction of the *μ*_a_ spectra of the kidney tissues, since for *λ* < 600 nm, hemoglobin presents absorption values at least 10× higher than lipids and 100× higher than water, as represented in Ref. [45].

After obtaining all the *μ*_a*i*_(*λ*) of the tissue components that were selected for the reconstruction of the *μ*_a_ spectra of both kidney conditions, it was necessary to obtain the optimal *w*_*i*_ values to use in Equation (2) to complete the reconstructions. To perform such a task, Equation (2) was rearranged, so that the least squares method could be used. Such a new arrangement, as presented in Equation (3) [22], was introduced in a MATLAB™ program based on the least squares algorithm to calculate the optimized *w*_*i*_ values that allow a good reconstruction of the experimental *μ*_a_ spectra of the kidney tissues.

(3) $$b={\left.(X^{T}X\right)}^{-1}\;X^{T}y$$

In Equation (3), *b* is a matrix that will contain the *w*_*i*_ values after calculation with the least squares algorithm, *X* is a matrix that contains the known *μ*_a*i*_(*λ*) of the tissue components that will be considered in the reconstruction procedure, and *y* is the mean experimental *μ*_a_(*λ*) of the healthy (or CRCC) kidney.

After calculating the optimal *w*_*i*_ values with the least squares algorithm for each condition of kidney, the reconstruction of the mean experimental *μ*_a_(*λ*) of healthy/CRCC kidney tissues was performed with Equation (2). Assuming that both kidney conditions contain a total of 77% of water, as reported for the healthy kidney [46], a final calculation was performed for each case to obtain the concentrations of the other chromophores from the *w*_*i*_ values that were estimated with the least squares method. This final calculation and the obtained results are presented in Section 3.

## 3 RESULTS AND DISCUSSION

This study was initiated with the spectral measurements. Using the setups presented in Figure 2, 10 *T*_t_ and 10 *R*_t_ spectra were acquired from the healthy kidney samples between 200 and 1000 nm. A similar number of spectra were collected from the CRCC samples. Using these 10 pairs of *T*_t_ and *R*_t_ spectra for each sample in Equation (1), 10 *μ*_a_(*λ*) were calculated for the healthy kidney, and another 10 *μ*_a_(*λ*) were calculated for the CRCC kidney.

**FIGURE 6:**
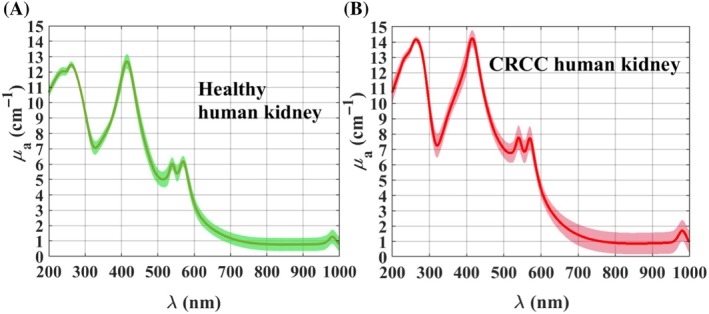
*μ*_a_(*λ*) spectra of healthy (A) and cancerous (B) human kidney.

To establish a statistically significant spectral reference of *μ*_a_ to be used in the reconstruction procedures, the mean and SD of the *μ*_a_(*λ*) of both kidney tissues were calculated and are presented in Figure 6.

The *μ*_a_(*λ*) curves presented in Figure 6 show valuable information to identify the components that both kidney tissues contain. These curves present absorption peaks located from the UV to the NIR, which can be associated with different tissue chromophores: proteins (~230 nm), DNA (~260 nm), hemoglobin (Soret band at ~415 nm, and Q‐bands at 540/570 nm) and water (980 nm) [45]. In this first analysis, no evidence of lipids was found in both tissues, although they were previously found in these two conditions of kidney tissues as a result of a tissue dispersion study [29].

Although both experimental *μ*_a_(*λ*) curves presented in Figure 6 show the same absorption peaks, the magnitude of those peaks is different between the healthy and CRCC kidney. Comparing between the absorption bands presented for the two kidney tissues, we see that the magnitude of all the absorption peaks is a little higher in the case of the CRCC kidney. This difference in peak magnitude indicates that the contents of proteins, DNA, blood, and water are higher in the CRCC than in the healthy kidney. Such information, although not too extensive, is according to previous studies, since it was already reported that tissues with cancer present higher contents of proteins and DNA [14], and a higher content of blood [12].

**FIGURE 7:**
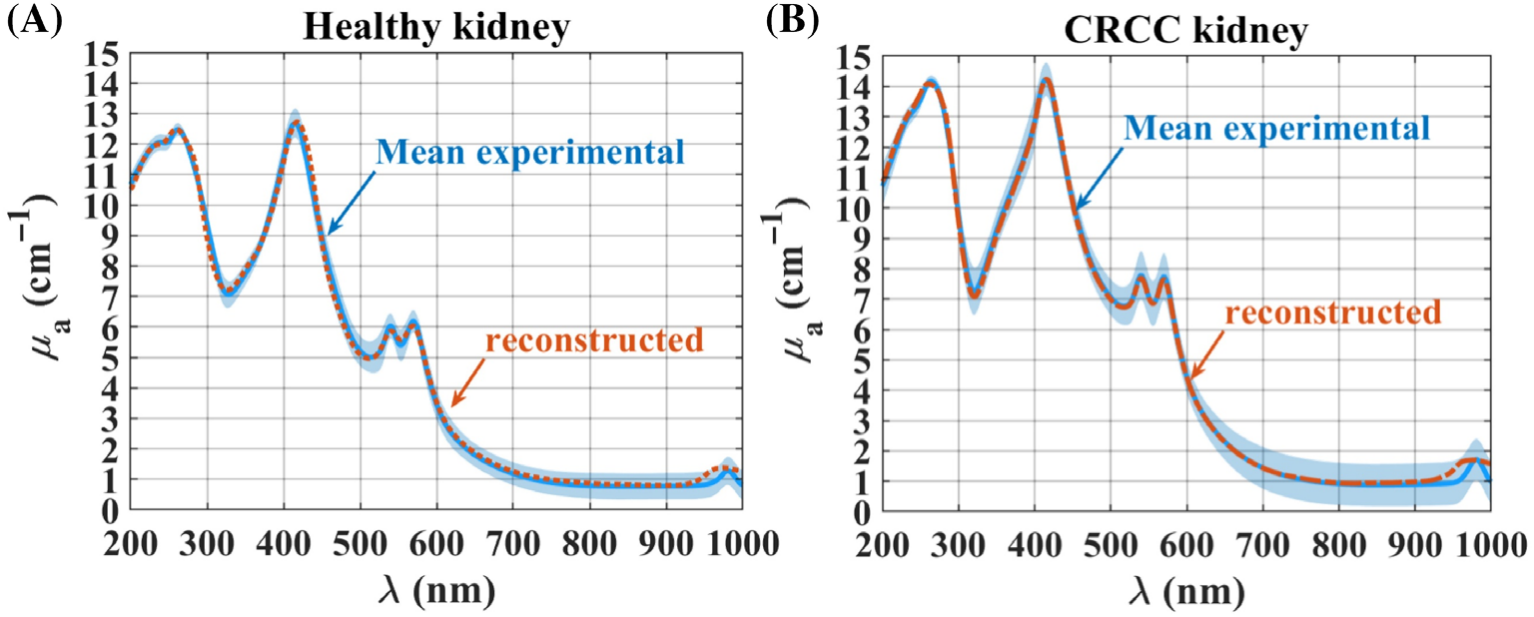
Mean experimental and reconstructed *μ*_a_ spectra of the healthy (A) and chromophobe renal cell carcinoma (CRCC) (B) human kidney.

To obtain additional diagnostic information, the reconstruction of the *μ*_a_(*λ*) of healthy and CRCC kidney were performed. Following the methodology described in Section 2.4, the least squares method was first used to obtain the optimal *w*_*i*_ values for each case. When such sets of values were obtained for both kidney conditions, Equation (2) was used to reconstruct both mean *μ*_a_ spectra. Figure 7 presents the same spectra as in Figure 6, and also the reconstructed spectra that were obtained.

According to Figure 7, a good matching between the mean experimental and the reconstructed *μ*_a_ spectra was obtained for both kidney conditions. These graphs show that the reconstructed spectra are always within the SD margins of the mean experimental *μ*_a_(*λ*), which confirms the good agreement. Only in the case of the absorption band of water (~980 nm), we observe a poor matching, where the reconstructed curve presents a broader band than the one seen in the experimental curve. The reason for this bad matching in the absorption band of water is that we used the absorption spectrum of pure liquid water in the reconstructions [42, 43, 44], where the water molecules are not bound to any other materials. In the case of tissues, water can be found in four states, where only one of those states is designated as free water: strongly bound, tightly bound, weakly bound, and free water [47, 48]. Other studies have reported that due to the water bounding to the other tissue components, the absorption peak at 980 nm can be narrower than the one for pure liquid water [49, 50]. This fact and also because 77% of the volume of kidney tissues is water [46], much of which is bounded water, explains why the absorption band seen in the experimental *μ*_a_(*λ*) of the kidney tissues is narrower than the one obtained in the reconstructed spectra.

To calculate the concentrations of the chromophores used in the reconstruction of the *μ*_a_(*λ*) of both kidney conditions, a simple conversion was considered. As mentioned in the previous paragraph, it has been reported that the healthy kidney of rats contains 77% of water [46]. Since no data for the human kidney has been reported, we assumed this value for both human kidney tissues. It is reasonable to assume the same water content for both the healthy and CRCC kidney since it has been reported that part of the bound water converts into mobile when cancer develops, keeping the total water content unchanged [12, 51]. Since we assumed that water represents 77% of the tissue volume for both kidney conditions, the other 23% correspond to the volume of the other tissue chromophores. Consequently, using this conversion rule, the concentrations of the other tissue chromophores that were considered in the reconstruction procedure were calculated for both kidney conditions. Table 1 contains the *w*_*i*_ values obtained from the least squares method and the calculated concentrations for each chromophore.

**TABLE 1:** Weights of the tissue chromophores used in the reconstruction of the *μ*_a_(*λ*) of the kidney tissues and calculated concentrations.

Type of kidney	Biological component/chromophore	Weight – *p*_i_	Concentration (%)
Healthy	Water	14.47	77.00
	Melanin	1.00	0.09
	Lipofuscin	5.65	0.51
	DNA	14.55	1.32
	HbO_2_	193.21	17.53
	Hb	37.28	3.38
	Proteins	1.71	0.16
	Lipids	0.07	0.01
		Total	100%
CRCC	Water	18.46	77.00
	Melanin	0.1	0.01
	Lipofuscin	8.97	0.66
	DNA	30.91	2.29
	HbO_2_	254.04	18.80
	Hb	11.05	0.82
	Proteins	5.22	0.39
	Lipids	0.43	0.03
		Total	100%

Abbreviations: CRCC, chromophobe renal cell carcinoma; Hb, hemoglobin; HbO_2_, oxygenated hemoglobin.

After obtaining the concentrations of all chromophores in the kidney tissues, it is now possible to compare between concentrations of each chromophore in the healthy and CRCC kidney. Regarding the pigments, the decrease observed for melanin from the healthy to the CRCC kidney is smaller than the observed increase of lipofuscin. These variations are in good agreement with the idea that lipofuscin and melanolipofuscin granules form with the development of cancer. We have assumed this idea in a previous work [29], and other authors have confirmed the formation of melanolipofuscin granules with the development of eye diseases [52]. In the case of DNA and HbO_2_, we see an increase from the healthy to the CRCC kidney. Such an increase is in good agreement with data from another study [14]. It is also observed from Table 1 that the concentration of Hb decreases from the healthy to the CRCC kidney. Finally, although we have not seen the presence of lipids as a separate band in the *μ*_a_ spectra presented in Figure 6, the reconstruction procedure was sensitive to their presence in both tissues. The data in Table 1 present that both kidney tissues have small amounts of lipids, but their concentration is higher in the CRCC kidney, as previously reported [29].

## 4 CONCLUSION

This study demonstrates that the *μ*_a_(*λ*) of any tissue can be reconstructed as a weighted sum of the individual absorption spectra of tissue components. By performing such reconstruction for the cases of human healthy and CRCC kidney, it was possible to obtain a good matching to the experimental *μ*_a_ spectra and evaluate different contributions of the tissue components in both kidney conditions, when spectra are measured from deep‐UV to NIR. Assuming that the total water content in healthy and CRCC kidney tissues represents 77% of tissue volume, allowed to calculate the concentrations of the other tissue components from the estimated contributions that were used in the reconstruction of the *μ*_a_ spectra. These concentrations showed that DNA, proteins, HbO_2_, and lipids have higher contents in the CRCC version of the kidney, a result that has previously been observed for other types of cancer. It was also observed that the concentration of lipofuscin increases from the healthy to the CRCC kidney in a higher extent than the observed decrease for the melanin concentration. Such a result is well in line with previous studies where similar variations were observed for other types of cancer and suggests the formation of melanolipofuscin granules as cancer develops. These results provide discriminating information for the case of CRCC and can be used for diagnostic purposes. To take benefit of the data collected in this study for future in vivo diagnostic protocols, our group plans to perform additional studies, where the minimally invasive measurement of *R*_d_ spectra will be used to develop ML algorithms to reconstruct the *μ*_a_ spectra of healthy and CRCC kidney tissues. In the case of human kidney, the measurement of *R*_d_ spectra can be performed in vivo with fiber endoscopes through an abdomen incision. When such algorithms are developed and validated, the reconstruction of the generated spectra can be made in a similar manner to the one in this study, so that the concentrations of the various tissue components can be evaluated for the establishment of a diagnostic.

## AUTHOR CONTRIBUTIONS

M.R.P. and L.E.F. were involved in the investigation and writing—original draft. V.V.T., L.M.O., and H.P.O. were involved in conceptualization, investigation, writing—review and editing. R.M.H., I.C.C., and S.D.C. were involved in investigation and writing—review and editing.

## CONFLICT OF INTEREST STATEMENT

The authors declare no financial or commercial conflict of interest.

## Data Availability

The data that support the findings of this study are available from the corresponding author upon reasonable request.
